# Ausência de Aerosclerose na Doença de Chagas: O Papel da Transialidase do Trypanosoma Cruzi

**DOI:** 10.36660/abc.20201229

**Published:** 2020-12-01

**Authors:** Maria de Lourdes Higuchi

**Affiliations:** 1 Universidade de São Paulo Instituto do Coração São PauloSP Brasil Universidade de São Paulo Instituto do Coração, São Paulo, SP - Brasil

**Keywords:** Doença de Chagas/fisiopatologia, Aterosclerose/fisiopatologia, Diagnóstico por Imagem/métodos, Tomografia Computadorizada Angiográfica

O artigo Menor Prevalência e Extensão da Aterosclerose Coronária na Doença de Chagas Crônica por Angiotomografia Cornária^1^ revela um dado muito importante: a população chagásica tem muito menos aterosclerose em comparação com uma população não chagásica cuidadosamente pareada. Analisando-se prospectivamente 43 pacientes, 93% dos pacientes chagásicos apresentavam ausência de placas de DAC, 7% de obstrução leve a moderada e nenhum caso de obstrução grave. O estudo endossa nossos dados patológicos anteriores^2^ em poucos corações autopsiados, confirmando que pacientes chagásicos não têm aterosclerose. Por que eles não apresentam aterosclerose?

A principal enzima produzida pelo *Trypanosoma cruzi* (T. cruzi) é a transialidase, que remove o ácido sálico do tecido para a circulação. A microbiota tem sido associada ao desenvolvimento da aterosclerose.^3^ O micoplasma é conhecido por se proliferar em meios ricos em colesterol e, em nosso estudo anterior, observamos grande quantidade de micoplasmas em placas de ateroma.^4^ Muitos agentes infecciosos como o micoplasma e vírus como o SARS CoV-2 usam o ácido sálico para entrar na célula hospedeira. A transialidase do *T. cruzi* pode remover os micoplasmas das placas de ateroma, evitando o desenvolvimento do ateroma.^5^ Criamos um nutricosmético associando a enzima transialidase a nanopartículas antioxidantes naturais e diminuição da aterosclerose experimental em coelhos.^6 , 7^

O presente trabalho enfatiza a necessidade de outra explicação além da DAC para a patogênese do infarto do miocárdio presente em pacientes chagásicos. Microinfartos, miocitólise, degeneração hialina e fibrose são achados comuns na cardiopatia chagásica crônica e têm sido atribuídos, em graus diversos, à miocardite crônica, fenômenos imunoalérgicos e alterações microvasculares.^8^ Observamos também que a artéria coronária direita distal era muito fina, associada ao adelgaçamento da parede ventricular, que pode ser interpretado como consequência da falta de pressão arterial intramiocárdica devido à microcirculação dilatada. Injetando-se 0,5% de nitrato de prata em solução aquosa de glicose a 5% para impregnar a superfície endotelial das artérias epicárdicas, arteríolas intramurais, foi possível observar que a microcirculação em autópsias de pacientes com doença de Chagas, a insuficiência cardíaca encontrava-se extremamente dilatada,^9^ possivelmente devido à inflamação miocárdica (pela presença de antígenos e simbiontes do *T. cruzi)* ^10 , 11^

Pode causar equilíbrio inadequado na distribuição do fluxo sanguíneo, pior perfusão tecidual em algumas áreas e múltiplos infartos. Por outro lado, as áreas fibróticas podem causar obstruções no trajeto dos vasos, favorecendo o desvio do fluxo sanguíneo (fenômeno de “roubo”) e aparecimento de lesões isquêmicas;^10^ as lesões de adelgaçamento características da doença chagásica nas paredes apicais e basais posteriores do ventrículo esquerdo também podem ser decorrentes de isquemia nas lesões nas áreas de *watershed* entre os dois ramos principais da artéria coronária — as artérias descendente anterior e descendente posterior, causando lesões isquêmicas, focos de infarto do miocárdio, aneurismas e fibrose miocárdica. A baixa perfusão na região de *watershed* das artérias coronária direita e circunflexa pode resultar em frequente lesão fibrótica de adelgaçamento da parede basal lateral do ventrículo esquerdo, levantando a hipótese de que essa lesão poderia ser um melhor preditora de taquicardia ventricular e morte súbita.^11^ Essa lesão miocárdica tem aspecto de cicatrização da infração miocárdica, contendo ilhas de miócitos viáveis em meio à fibrose, podendo induzir arritmia ventricular.^12^

A cardiopatia chagásica é uma doença peculiar que ainda demanda muitas pesquisas.
