# Supplementary material for: Ausência de Aerosclerose na Doença de Chagas: O Papel da Transialidase do Trypanosoma Cruzi
Source: Arq Bras Cardiol. 2020 Dec 1;115(6):1061–2. [Article in Portuguese] doi: 10.36660/abc.20201229 (PMC8133730; doi:10.36660/abc.20201229)
Supplement: Translation

# Translation: Absence of Atherosclerosis in Chagas’ Disease: The Role of Trypanosoma Cruzi Transialidase

**Authors:** Maria de Lourdes Higuchi

**Affiliations:** 1 Brazil Universidade de São Paulo Instituto do Coração, São Paulo, SP - Brazil

**Keywords:** Chagas Disease/physiopathology, Atherosclerosis/physiopathology, Diagnostic Imaging/methods, Computed Tomography/methods

The paper Lower Prevalence and Severity of Coronary Atherosclerosis in Chronic Chagas’ Disease by Coronary Computed Tomography Angiography^1^reveals a very important data: patients with Chagas disease have much lower atherosclerosis compared with a carefully matched population of individuals without Chagas disease. Analyzing 43 patients prospectively, 93% of Chagas disease patients had absence of coronary artery disease (CAD) plaques, 7% mild to moderate obstruction and zero cases of severe obstruction. It endorses our previous pathological data^2^in few autopsy hearts, confirming that Chagas disease patients do not have atherosclerosis. Why they do not present atherosclerosis?

The main enzyme produced by *Trypanosoma cruzi* ( *T. cruzi* ) is trans-sialidase. It removes syalic acid from the tissue to the circulation. Microbiota has been associated with the development of atherosclerosis.^3^Mycoplasma is known to grow in cholesterol-rich media and in our previous study we observed large amounts of mycoplasmas in fat atheroma.^4^Many infectious agents such as mycoplasma and viruses such as SARS CoV-2 uses the syalic acid to enter the host cell, the transialidase from *T. cruzi* may remove mycoplasmas from the atheroma plaques, preventing development of atheroma.^5^We created a nutricosmetic associating the enzyme transialidase and natural antioxidant nanoparticles and decreased experimental atherosclerosis in rabbits.^6 , 7^

The present study emphasizes the need of other explanation than CAD for myocardial infarction pathogenesis present in Chagas disease patients. Microinfarcts, myocytolysis, hyaline degeneration and fibrosis are common findings in chronic Chagas disease cardiopathy and have been attributed, in varying degrees, to chronic myocarditis, immunoallergic phenomena and microvascular alterations.^8^We also observed that distal right coronary artery was very thin, associated with ventricular wall thinning, which may be interpreted as a consequence of lack of intramyocardial blood pressure due to dilated microcirculation. Injecting 0.5% silver nitrate in 5% aqueous glucose solution to impregnate the endothelial surface of the epicardial arteries and intramural arterioles, it was possible to see that microcirculation in autopsies of patients with Chagas disease heart failure was extremely dilated,^9^possibly due to myocardial inflammation (due to the presence of *T. cruzi* antigens and symbionts).^10 , 11^

It may cause inadequate balance in the blood flow distribution, worst tissue perfusion in some areas and multiple infarctions. On the other hand, the fibrotic areas may cause obstructions in the vessel trajectory, favoring deviation of blood flow (a “steal” phenomenon), and appearance of ischemic lesions;^10^the characteristic thinning lesions in Chagas disease at the apical and basal posterior left ventricle walls may also be the result of ischemia in the “watershed” lesions between the two main coronary artery branches — the anterior descending and posterior descending arteries causing ischemic lesions, foci of myocardial infarction, aneurysms and myocardial fibrosis. Low perfusion in the watershed region of right coronary and circumflex arteries may result in frequent thinning fibrotic lesion of lateral basal left ventricular wall, raising the hypothesis that this lesion could be a better predictor for ventricular tachycardia and sudden death.^11^This myocardial lesion has an aspect of myocardial infarction healing, containing islands of viable myocytes in the midst of fibrosis, which may induce ventricular arrhythmia.^12^

Chagas disease cardiopathy is a particular disorder that still deserves many research studies.
